# Psychological Capital and Career Commitment Among Chinese Urban Preschool Teachers: The Mediating and Moderating Effects of Subjective Well-being

**DOI:** 10.3389/fpsyg.2021.509107

**Published:** 2021-07-22

**Authors:** Yongtao Gan, Li Cheng

**Affiliations:** ^1^School of Law, Shantou University, Shantou, China; ^2^School of Education, Hubei University Education College, Wuhan, China

**Keywords:** preschool teachers, career commitment, psychological capital, subjective well-being, mediating effect, moderating effect

## Abstract

This study explored the effects of psychological capital (PsyCap) on career commitment among preschool teachers in China, with a particular focus on the mediating effects of subjective well-being (SWB). A total of 759 teachers were subjected to the PsyCap Questionnaire and Career Commitment Scale. The data were analyzed and used for structural modeling with Mplus Version 7.4. Results indicated that PsyCap positively influenced career commitment, with SWB significantly mediating and moderating this causal association. Thus, the influence of PsyCap on career commitment is improved through enhanced SWB. These findings highlight avenues for improving both PsyCap and career commitment in the Chinese context among urban preschool teachers.

## Introduction

As professionals in early childhood education and care, preschool teachers are important human resources. Their influence on child development is recognized in a worldwide context, but the early childhood education system can create a stressful environment (Zhai et al., 2011). Nevertheless, many preschool teachers faithfully attend to their duties and actively dedicate themselves to the educational field. This likely takes substantial psychological capital (PsyCap), which is a concept derived from positive psychology (i.e., a focus on healthy behaviors rather than dysfunction). It is a kind of positive psychological state in which individuals can process information and develop (Luthans et al., 2007). Further, PsyCap can effectively be used to regulate individual psychological states and emotions, guide employees in producing effective organizational attitudes and positive behaviors, and facilitate positive responses to environmental changes. Indeed, previous studies have found that PsyCap can help preschool teachers increase their work inputs while reducing job burnout and buffering occupational pressures (Rentzou, 2012).

As they are faced with many difficulties, preschool teachers need positive psychology to create beneficial emotional states. In this context, positive PsyCap can enhance work attitudes and behaviors. Career commitment (CC) refers to an individual’s attitude toward their career, including their willingness and enthusiasm to work in a given capacity. Previous studies have found that CC leads to better performance regarding both focal and discretionary behaviors (Tsoumbris and Xenikou, 2010) and reduces job turnover rates (Kim, 2018). Further, high levels of CC help teachers provide better childcare and education (Lee et al., 2017). The main premise underlying self-determination theory (SDT) is that people are naturally inclined to be industrious and build an expanded and consolidated feeling of self (Sheldon et al., 2003). That is, they search for ways to connect with themselves, other persons, and groups in the community. Additionally, when the needs of autonomy, competence, and sense of belonging are fulfilled, individuals’ well-being and social progress are advanced.

When experiencing positive psychological states and high levels of CC, individuals are more likely to work under pressure or other adverse circumstances and may show greater dedication to their work over the course of a career (Rentzou, 2012). In this context, subjective well-being (SWB) is an important predictor for one’s level of CC. Further, employees with better PsyCap tend to have both better SWB and increased CC (Singhal and Rastogi, 2018). This implies that individuals with high PsyCap experience more positive emotions and have greater work satisfaction, which can further improve SWB, while alleviating or reducing energy consumption and enhancing CC. As such, PsyCap can improve CC by enhancing SWB, which may thus have a mediating effect on the relationship between PsyCap and CC.

Previous research has found that SWB may play a functional role in promoting lifelong success (Diener, 2008), meaning that SWB is not merely a result of success, but may also impact other related aspects. For instance, SWB influences the individual’s decision-making process, enhances physical health, and facilitates stress coping by inducing positive emotions; thereby, clarifying problems and helping to generate a variety of solutions (Brickman et al., 1978). SWB can regulate and/or cushion stress by inducing positive psychological states, adjusting perspectives, and either enhancing or weakening career attitudes (e.g., De Neve and Oswald, 2012; Stansfeld et al., 2013). We therefore suggest that PsyCap and CC are moderated by SWB. As such, this study investigated the association between PsyCap and CC with a focus on the mediating and moderating effects of SWB among preschool teachers.

The broaden and build theory posits that positive emotions promote a person’s reasoning and behavior to a new prospect (Fredrickson, 2004). This improves well-being due to an increase in physical, psychological, and social resources (Fredrickson, 2004). Positive emotions, such as satisfaction, happiness, and love, broaden people’s thought action pool, negate negative emotions, enhance resilience, expand resources, and further lead to improved contentment. Additionally, SDT postulates that autonomy, competence, and relatedness lead to well-being (Ryan and Deci, 2000). This theory posits that engaging in an activity willingly and inner satisfaction leads to fulfilling the need of autonomy. Moreover, by fulfilling an arduous task successfully and showing one’s talent and ability, one’s competency need can be met. To achieve a sense of relatedness, getting close to one’s loved ones and having purposeful conversations can help achieve this need. SDT suggests that a combination of these three psychological needs will promote people’s health and development, as well as their potential, and enhance their sense of happiness. As predicted by SDT, one study found that “good days” are positively correlated with the realization of these three requirements (Reis et al., 2000). In the present study, we will use the tenets of these two theories to build our hypotheses.

Psychological capital refers to four aspects of the personal positive psychological status, including self-efficacy, optimism, hope, and resilience (Youssef and Luthans, 2007). Many previous studies have found that PsyCap plays positive roles in individual and organizational development. Further, there is an established association between PsyCap and CC (Singhal and Rastogi, 2018). Enhanced PsyCap can thus reduce employee turnover rates (Allameh et al., 2018), help establish organizational identities through employee support, enhance individual identities within the organizational context, promote organizational commitment (Luthans et al., 2007), and reduce both voluntary absenteeism and involuntary absenteeism (Avey et al., 2006).

Research has demonstrated a significant and positive association between PsyCap and organizational commitment. Specifically, activities that elevate PsyCap promote organizational commitment (Sharifi and Shahtalebi, 2015). PsyCap is also a significant predictor of CC, with the latter increasing alongside the former (Sinan, 2016; Lee et al., 2017). Recently, theoretical developments have revealed that improved positive PsyCap can lead to better overall organizational commitment, favorable organizational citizenship behaviors, lower rates of employee absenteeism, and higher job satisfaction (Tian and Zou, 2013; Idris and Manganaro, 2017). Therefore, the following hypothesis is proposed.

*H1*: PsyCap is positively associated with CC.

Subjective well-being is a central concept of both humanistic psychology and positive psychology. It specifically refers to “people’s cognitive and affective evaluations of their lives” (Diener, 2000). In this context, the term “subjective” refers to the inherent views of respondents without enforcing any unrelated reference frame. As such, it is used to measure individual-based steady well-being rather than temporary emotions (Singhal and Rastogi, 2018).

The foundation of this connection is that PsyCap includes the personal characteristics that affect people’s SWB, which enables people to achieve personal development, autonomy, a purposeful life, and helpful relationships (Cole et al., 2009).

According to the broaden and build theory, positive emotions increase a person’s sense of happiness by establishing physical, psychological, and social resources (Fredrickson, 2004). In this sense, teachers’ sense of PsyCap imparts innovation and emotional intellect (Salovey et al., 2002), which improves SWB in terms of personal psychological capacity and strength (Lewis et al., 2011). Moreover, teachers’ well-being and the consequent status of the labor market are influenced by the teachers’ PsyCap (Dockery, 2016). This suggests that it does not matter whether people are employed, but that individual well-being will contribute to one’s PsyCap. Therefore, the worse the PsyCap, the worse an individual’s well-being. Therefore, the following hypothesis is proposed.

*H2*: PsyCap is positively associated with SWB.

While many studies have independently examined SWB and CC, few have explored the relationship between the two. Further, most of these have concentrated on the intersection between organizational commitment and well-being (Sawada, 2013). For instance, Meyer et al. (2012) developed a theoretical framework showing that well-being predicted commitment outcomes. Other studies have suggested that well-being is a predictor of turnover intentions (Brunetto et al., 2013) and that CC can effectively reduce them. As such, we posit that there is a direct relationship between SWB and CC. This further implies that SWB plays a mediating role in the relationship between PsyCap and CC. In the context of this study, such a relationship is also believed to apply to preschool teachers. Therefore, the following hypothesis is proposed.

*H3*: SWB is positively associated with CC.

Further, implicit well-being has a moderating (or buffering) effect on stress. Lightsey (1994) found that an abundance of self-worth could create positive unconscious thinking, which may in turn cushion the pressure of negative emotional states. Positive unconscious thinking in persons experiencing strong happiness can help them buffer the connections between negative life events, negative thoughts, and negative adaptation results. SWB generally influences perspectives, coping methods, and the mental approaches to problems and/or stressors by inducing positive emotions.

People with higher levels of happiness tend to exhibit more positive emotions, such as enthusiasm, helpfulness, optimism, and satisfaction, as well as fewer negative emotions (Withey et al., 2005). A study by Guven (2011) confirmed these findings. People with high levels of happiness are also more willing to trust others and participate more actively in social activities, thus maximizing PsyCap. Notably, Graham et al. (2004) found that happiness was related to both future income and health. Further, urban residents with high levels of happiness tend to have more optimistic attitudes and participate more fully in the labor market (Graham et al., 2004). People with high SWB are also more likely to gain greater economic independence, have higher hopes for the future, and have better long-term physical fitness (Guven, 2012).

As SWB substantially influences individuals’ emotions and behaviors, encouraging SWB is likely to help promote the positive impacts of PsyCap among preschool teachers. When compared to individuals with low SWB, the predictive effects of PsyCap on CC may be more obvious among those with high SWB.

Few studies have directly examined the mediating effects of the mechanisms which support CC through the underlying mechanism which controls SWB. However, there is growing evidence that individuals with high SWB experience higher levels of CC. Still, few studies have organically combined these elements to analyze their impacts on CC. Most notably, the potential moderating effects of SWB remain largely unexamined. As such, this study combined PsyCap and SWB to understand the impact of SWB on CC, focusing on both the mediating and moderating effects of SWB. Therefore, the following hypothesis is proposed.

*H4*: SWB positively moderates the relationship between PsyCap and CC.

Although different studies have either directly or indirectly found that PsyCap changes the focus of CC, none have explored its internal mechanism (especially the mediating effects and conditions under which PsyCap will enhance or weaken certain characteristics). Diener (2008) proposed that SWB can promote success in all areas of life. Such functionality is mainly achieved through the “broaden-build” effects of positive emotions (Fredrickson, 1998). Here, broaden refers to the broadening of individual cognition; positive emotions can help individuals break certain restrictions and stimulate new ideas, thus expanding both the scope of individual attention and the instantaneous thinking-action ability (Fredrickson, 2001). The building function, meanwhile, refers to how positive emotions can help individuals discover and develop their own strengths, increase the flexibility of individual behaviors, and actively construct lasting biological-psychological capital (Fredrickson and Branigan, 2005). SWB among preschool teachers will differ based on the PsyCap conditions (Hall-Kenyon et al., 2014). Due to this, some individuals will have different judgments even when operating on the same levels of PsyCap. Moreover, because of their differing degrees of professional recognition and subjective initiative, PsyCap in preschool teachers will inevitably have an impact on their career commitment. Therefore, the following hypothesis is proposed.

*H5*: SWB positively mediates the relationship between PsyCap and CC.

**Figure 1:**
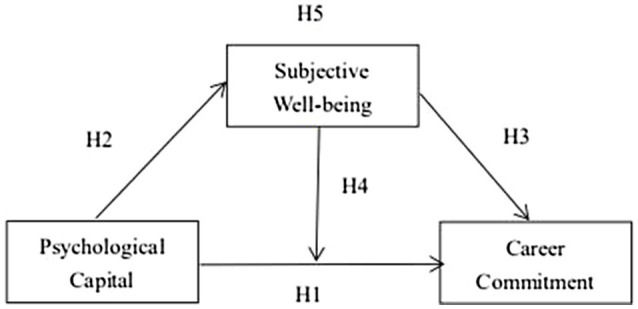
The concept model.

Hence, based on the above theoretical basis and hypothetical deduction, this paper proposes a conceptual model for Studies 1 and 2, which are shown in Figure 1.

## Materials and Methods

This study quantitatively explored how SWB mediated and moderated the relationship between PsyCap and CC among urban preschool teachers in the capital city of Wuhan in Hubei Province, China. This descriptive and correlational study was conducted to assess the main variables as they related to preschool teachers. As such, the target research population included 759 preschool teachers in Wuhan. This number was determined by data obtained from a survey conducted among 41 preschools in Wuhan. All 759 preschool teachers completed and submitted questionnaires during the data collection period (March 10 to June 20, 2019).

### Participants

A total of 759 preschool teachers were random sampling from 26 urban preschools in Wuhan city, Hubei Province, China. Of these, 751 (98.95%) teachers were female and eight (1.05%) were male. The mean age was 33, with a standard deviation of 5.25. Regarding their academic titles, 59 (7.77%) were considered seniors, 136 (17.92%) were intermediates, 202 (26.61%) were juniors, and 362 (98.95%) were unrated; 569 (47.69%) taught at public institutions, while 190 (25.03%) taught at private locations. Further, 402 (52.96%) taught demonstration preschool classes, while 126 (16.60%) taught first-class preschool; 117 (15.42%) taught second-class preschool; and 114 (15.02%) taught third-class preschool. Survey data revealed that 377 (49.67%) of the teachers earned less than CNY 2,500 per month, 223 (29.38%) earned CNY 2,500–4,000 per month, and 158 (20.82%) earned more than CNY 4,000 per month.

### Data Collection Technique and Tools

An electronic link to the questionnaire was established after corresponding with the Hubei Academy of Education. The preschool Education Monitor then sent this link to the e-mail addresses of all eligible teachers in Wuhan. Participants accessed an informed consent form before answering any questionnaire items. As such, they were only able to access the questionnaire after providing consent. All completed informed consent forms, and questionnaires were automatically sent to this researcher’s e-mail address. Of the 949 preschool teachers invited to participate, 759 valid forms were collected.

The questionnaire was comprised of the Social Demography and Personal Information (SDPI) Scale, Psychological Capital Questionnaire (PCQ), Subjective Well-Being Scale, and Career Commitment Scale. The SDPI consisted of 13 items (including those on age, gender, marital status, and education attainment) to obtain descriptive characteristics related to sociodemographic and job variables.

#### Psychological Capital Questionnaire

We assessed PsyCap according to the PCQ which was compiled by Luthans et al. (2007) and translated into Chinese by Li (2008). This scale comprises 24 items which were categorized into four subdimensions, including hope (six items), self-efficacy (six items), optimism (six items), and resilience (six items); items designed to assess negative attitudes were calculated using reverse scoring. Higher overall scores indicated higher PsyCap. The internal consistency coefficient of PsyCap (Cronbach’s alpha) was 0.95 (with values between 0.87 and 0.94 for the subscales).

#### Subjective Well-Being

Subjective well-being was assessed according to the Subjective Well-Being Scale for Chinese Citizen (Brief Edition; Xing, 2003). This consisted of 20 items across the 10 dimensions, two items per dimension, including contentment and abundant experience (sample item, “As I grew older, I learned a lot from my life, which made me stronger and more capable”), psychological health (sample item, “When it comes to unhappy things, I cannot cheer up for a long time”), social confidence (sample item, “Society will provide more and more outlets for people”), growth and progress (I am glad that my views have become more and more mature over the years), importance of goals (sample item, “Most of the life goals I set can give me encouragement rather than frustration”), self-acceptance (sample item, “I often feel uncomfortable in some parts of my body”), physical health (sample item, “I’m very upset about my health”), psychological balance (sample item, “Compared with the people around me, I am content”), interpersonal adaptation (sample item, “I often find it difficult to build friendships with others”), and family atmosphere (sample item, “I sometimes find it difficult to communicate with my family”). All items were rated according to a 7-point scale ranging from 1 (Never) to 7 (Always). Higher scores on this scale were associated with increased SWB levels. We used confirmatory factor analysis (CFA) to test SWB. *χ*^2^/df = 2.94, CFI = 0.96, RMSEA = 0.058 [0.036, 0.075], and SRMR = 0.029 [0.018, 0.043]. The internal consistency coefficient of SWB (Cronbach’s alpha) was 0.93.

#### Career Commitment Scale

CC was assessed according to a scale designed by Meyer and Allen (1991). It consisted of nine total items across the three subdimensions of affective commitment (AC), continuance commitment, and normative commitment (NC). All items were answered according to a 7-point Likert scale ranging from 1 (Never) to 7 (Always). The overall internal consistency coefficient was determined as 0.87 (with values between 0.80 and 0.85 for the subscales). Higher overall scores indicated higher CC.

#### Control Variables

Participants reported their ages, education attainment levels (ranging from 1 = “some high school” to 5 = “graduate degree”), salary, marital status, teaching years, teaching types (0 = “temporary preschool teacher” and 1 = “regular preschool teacher”), and school type (0 = “private” and 1 = “public”).

### Data Analysis

We compute polychoric correlation matrix with using IBM SPSS 21.0. This was done to check construct validity for each dimension according to the item-to-scale correlations (Table 1). As seen, good correlations were found among all variables.

**Table 1:** Item-to-scale correlation for the main variables.

S. No.	Variable	1	2	3	4	5	6	7	8
1	Self-efficacy	–							
2	Hope	0.815^**^	–						
3	Resilience	0.488^**^	0.612^**^	–					
4	Optimism	0.525^**^	0.642^**^	0.561^**^	–				
5	Subjective well-being	0.381^**^	0.528^**^	0.501^**^	0.570^**^	–			
6	Affective commitment	0.390^**^	0.545^**^	0.446^**^	0.500^**^	0.530^**^	–		
7	Continuance commitment	0.345^**^	0.543^**^	0.490^**^	0.455^**^	0.535^**^	0.587^**^	–	
8	Normative commitment	0.410^**^	0.559^**^	0.454^**^	0.496^**^	0.549^**^	0.634^**^	0.604^**^	–
	Mean	4.33	4.57	4.64	4.66	3.63	4.08	3.56	3.78
	SD	1.06	0.88	0.83	0.84	0.60	0.64	0.85	0.85

^*^P < 0.05;

** *P < 0.01*.

Therefore, the correlation results suggested that correlations between variables were consistent with the research hypotheses; thus, all hypotheses were tested through structural equation modeling with Estimator ML Optimization method by using Mplus (7.4). Although no significant chi-square statistics showed good fit, most models were expected to have significant chi-square statistics due to the relatively large survey sample. As such, additional fitting indices were also applied. Here, CFI values greater than 0.95 and RMSEA values less than 0.05 indicated good model fit (Kline, 2011). The significance level for all assessments was set at *p* < 0.05. A power analysis indicated that the proposed analysis models could be used to test the hypotheses.

We further explored how SWB moderated the relationship between PsyCap and CC through structural equation modeling. As a moderating variable, SWB referred to the different levels of effect that PsyCap had on CC among urban preschool teachers. There are many ways to test the effects of such regulation. However, this study conducted a partial regression coefficient test of both the independent and regulatory variables. The first step involved a multiple regression using PsyCap and SWB as independent variables with CC as the dependent variable. The second step involved testing the partial regression coefficients of PsyCap and SWB. Here, the moderating effects were obvious because the partial regression coefficient was significant.

**Figure 2:**
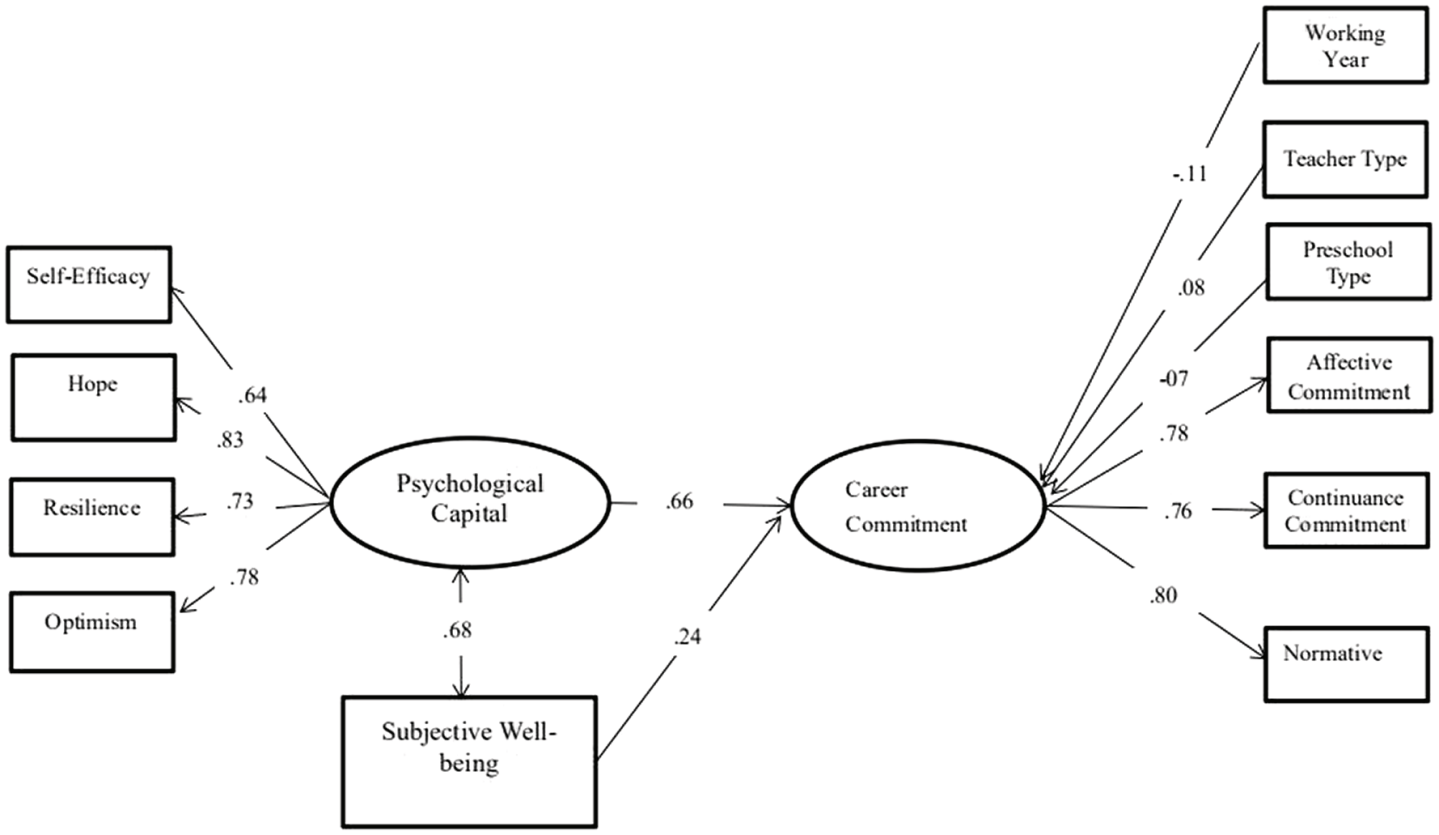
Associations between psychological capital (PsyCap), subjective well-being (SWB), and occupational commitment (*N* = 759).

**Figure 3:**
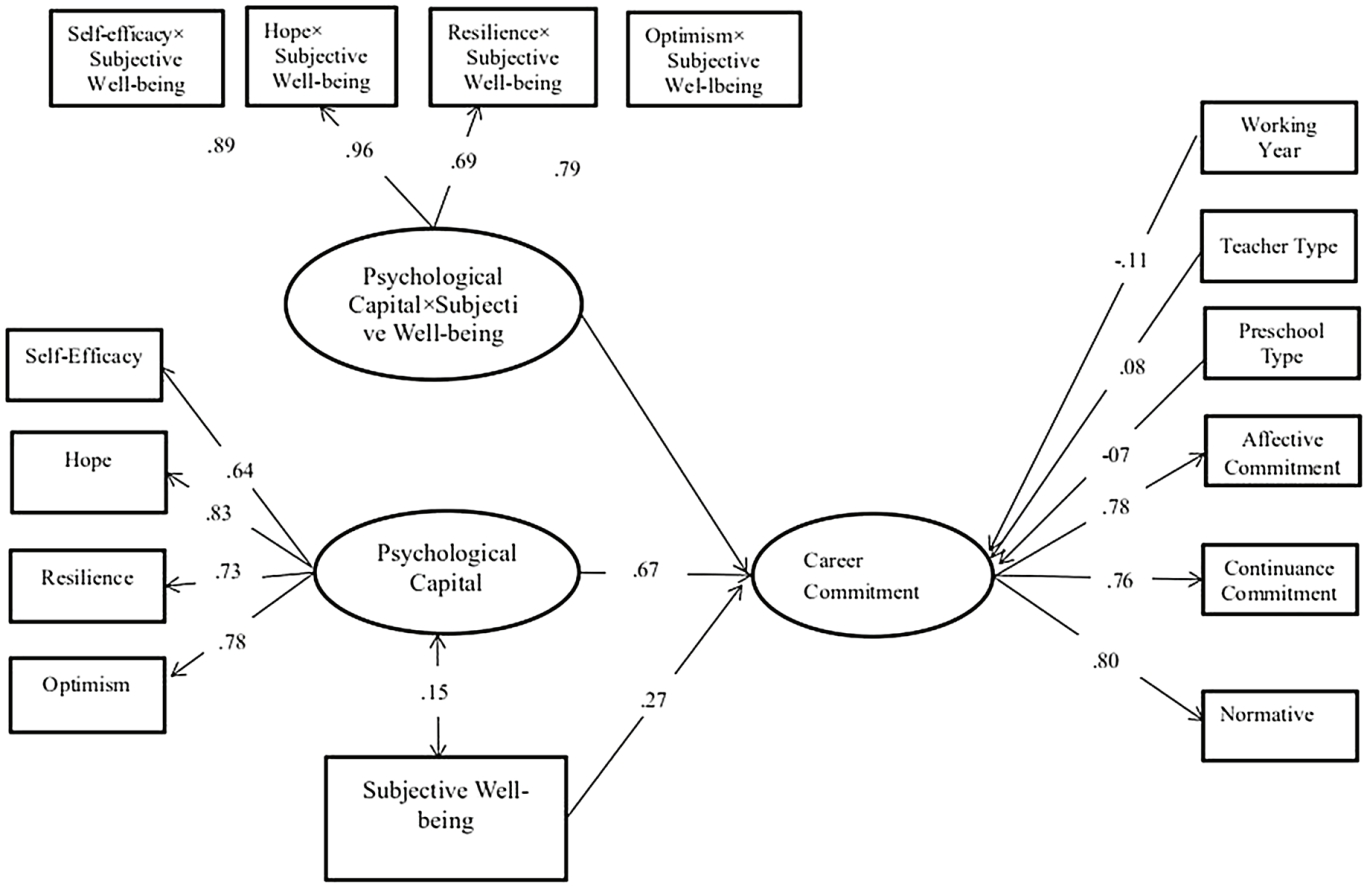
Associations between PsyCap, SWB, and career commitment (*N* = 759). Significant standardized coefficients were presented. Nonsignificant coefficients were not presented.

A total of three structural models were examined. In the first, PsyCap and SWB were specified as exogenous variables that predicted CC (Figure 2). The seven variables of age, education attainment level, salary, marital status, working year, teaching type, and preschool type were used as controls. A bootstrap approach was implemented in the second model (Figure 3). The seven above mentioned variables were specified as exogenous control variables linked to CC. PsyCap × SWB was examined in the second model (Figure 3). The interactions between these two factors were examined to determine the potential buffering effects of SWB. This is a valid and powerful method for testing mediating effects due to its resampling strategy in calculating mediating effects while making no assumptions about the shape of the coefficient sampling distribution (Preacher et al., 2007). If a significant interaction emerged, then the simple slopes were examined to assess the association between PsyCap and CC at the +1 SD and −1 SD of the moderator (i.e., SWB).

## Results

### Direct Effects Model

To test the impact of PsyCap (the latent variable), SWB (the manifest variable) on CC (the latent variable) was determined. The path model showed good fit on the research data, and the results are presented in Figure 2. Results were *χ*^2^ = 193.591, *p* < 0.01; CFI = 0.97; RMSEA = 0.047, 90% CI [0.039, 0.055] for the model examining the associations between PsyCap, SWB, and CC (Figure 2). Here, SWB was associated with CC in the expected directions after controlling for the seven above mentioned demographic variables. The main effects of the three CC dimensions and four PsyCap dimensions were simultaneously evaluated to determine which dimension was correlated with preschool teacher responses in a single given model. The CC model was determined as adequate at *χ*^2^ = 1, *p* > 0.1; CFI = 0.99; RMSEA = 0.007, while the PsyCap model was adequate at *χ*^2^ = 0.393, p > 0.1; CFI = 0.99; RMSEA = 0.008.

As shown in Figure 2, PsyCap had a significant positive impact on CC (*β* = 0.66, *p* < 0.001); thus, H1 was verified. We also carried out the same test on the effects of SWB on CC. The results indicated that SWB had significant positive impacts on CC (*β* = 0.24, *p* < 0.001). Therefore, H2 was also supported.

### Subjective Well-Being as a Buffer: The Interaction Between PsyCap and CC

For the models examining the interaction effects between PsyCap (the latent variable) and SWB (manifest variable), a latent interaction term was observed after multiplying the four PsyCap indicators with the SWB values. The model fits the data well at *χ*^2^ = 216.456, *p* < 0.01; CFI = 0.98; RMSEA = 0.061, 90% CI [0.053, 0.073]. After entering PsyCap × SWB, the effect of PsyCap on CC (*β* = 0.67, *p* < 0.001) and the effect of SWB on CC increased (*β* = 0.27, *p* < 0.001). Therefore, PsyCap significantly interacted with SWB in predicting CC (Figure 3). The interactive effects were statistically significant (*β* = 0.11, *p* < 0.001). Consistent with our hypotheses, these results suggested that SWB effectively moderated the relationship between PsyCap and CC. Thus, H4 was supported.

### Psychological Capital Dimensional Interactions With SWB and Predicting the CC Dimension

**Figure 4:**
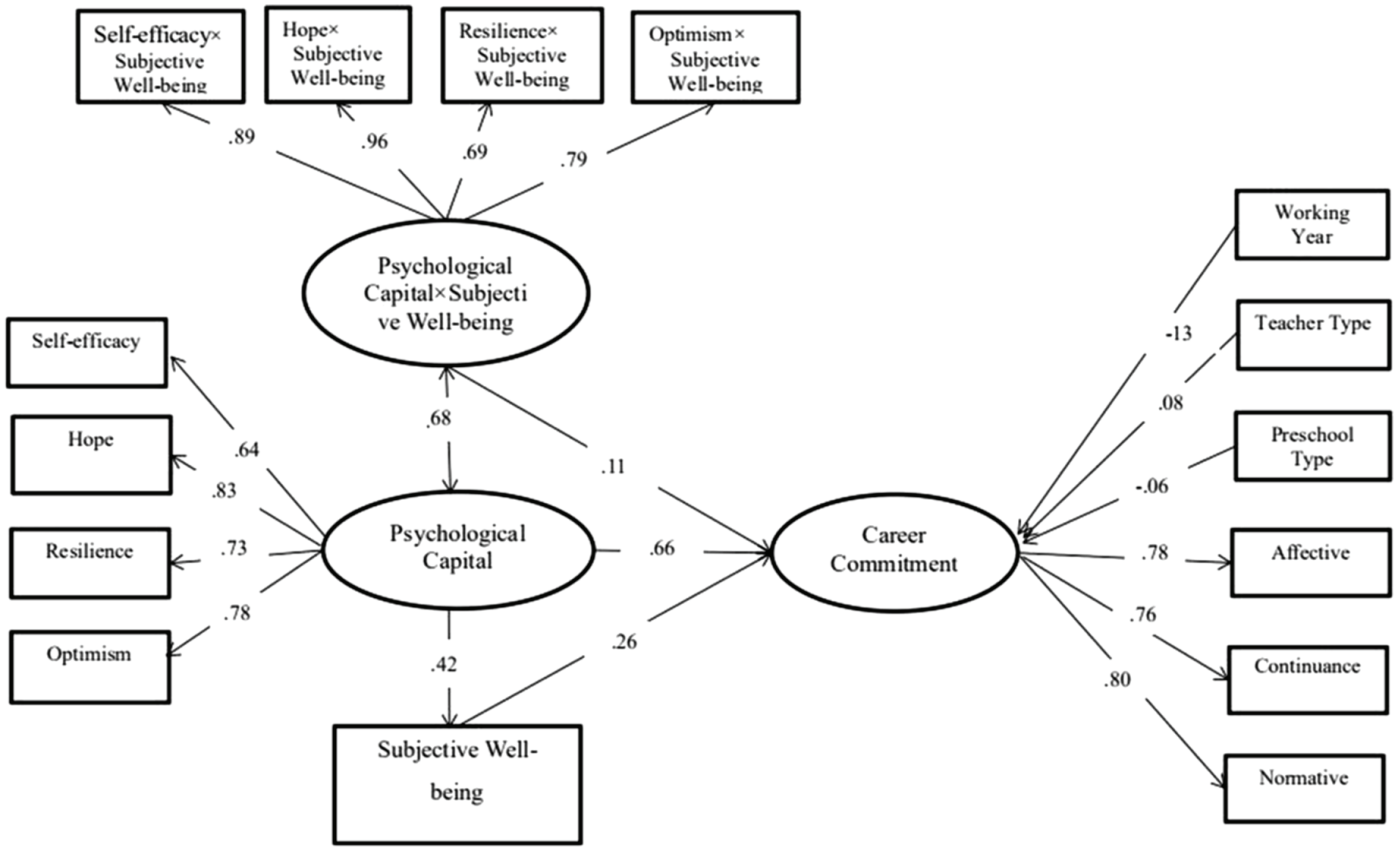
The mediating and moderating effect of SWB on the relationship between PsyCap and career commitment (*N* = 759). Significant standardized coefficients were presented. Nonsignificant coefficients were not presented.

The moderating effect of SWB was examined using a simple slope test to more clearly determine the essence of the interaction effect. Here, the subjects were divided into high and low groups with the upper and lower standard deviations of SWB among participants set as the boundary. The interaction model involving the four dimensions of PsyCap × SWB also indicated good fit at *χ*^2^ = 63.382, *p* < 0.01; CFI = 0.98; RMSEA = 0.078, 90% CI [0.066, 0.097]. The interaction effects for PsyCap with SWB predicting CC are shown in Figure 4. Specifically, PsyCap was significantly associated with CC when SWB was either low (−1 SD, *b* = −0.124, *β* = 0.57, *p* < 0.001) or high (1 SD, *b* = 0.32, *β* = 0.75, *p* < 0.001; Figure 4). These results indicate that both the low and high SWB groups significantly trended upward in CC with increased PsyCap. To further explain the moderating effect of SWB, we plotted schematic diagrams as shown in Figure 4.

To further explore the moderating effect of SWB between the four dimensions of PsyCap and three dimensions of CC, we adopted the PROCESS 3.3 module to analyze the significance of specific moderating effects. The interaction model involving SWB was also of good fit; six significant interactive effects were identified (Table 2). First, the two PsyCap dimensions of self-efficacy and optimism interacted with observed SWB in predicting AC (Table 2). Observed hope and resilience, however, were not associated with SWB in predicting AC; observed resilience was also not associated with SWB in predicting the other two CC dimensions. Second, the three PsyCap dimensions of hope, self-efficacy, and optimism interacted with observed SWB in predicting NC (Table 2). Observed resilience, however, was not associated with SWB in predicting any of the three CC dimensions. Third, only observed optimism interacted with observed SWB in predicting continuance occupation (Table 2).

**Table 2:** The predictive effect of psychological capital on career commitment at different levels of subjective well-being (SWB).

PsyCap dimensions interactions with SWB	SWB	*b*	*β*	LLCI	ULCI
Hope interacted with SWB predicting affective commitment	M−SD	0.08	0.32^***^	0.24	0.40
	M	0.22	0.42^***^	0.35	0.49
	M+SD	0.46	0.49^***^	0.41	0.58
Self-efficacy interacted with SWB predicting affective commitment	M−SD	0.18	0.20^***^	0.10	0.31
	M	0.18	0.31^***^	0.24	0.42
	M+SD	0.54	0.42^***^	0.31	0.53
Hope interacted with SWB predicting normative commitment	M−SD	0.22	0.28^***^	0.21	0.36
	M	0.04	0.38^***^	0.33	0.46
	M+SD	0.32	0.48^***^	0.40	0.56
Self-efficacy interacted with SWB predicting normative commitment	M−SD	0.32	0.20^***^	0.10	0.30
	M	0.03	0.31^***^	0.22	0.39
	M+SD	0.37	0.41^***^	0.31	0.51
Optimism interacted with SWB predicting normative commitment	M−SD	0.30	0.25^***^	0.16	0.34
	M	0.03	0.34^***^	0.25	0.42
	M+SD	0.28	0.40^***^	0.31	0.50
Optimism interacted with SWB predicting continuance commitment	M−SD	0.55	0.25^***^	0.14	0.36
	M	0.14	0.32^***^	0.22	0.42
	M+SD	0.16	0.37^***^	0.26	0.49

^*^P < 0.05;

^**^P < 0.01;

*** *P < 0.001*.

### The Mediating and Moderating Effects of SWB on Relationship Between on PsyCap and CC

**Figure 5:**
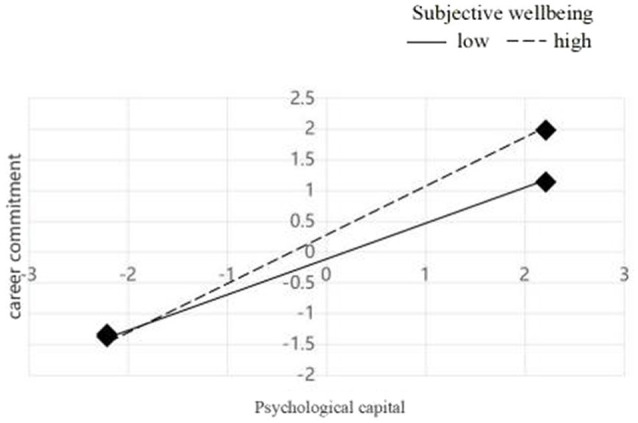
PsyCap interacting with SWB and predicting career commitment (*N* = 759).

The moderated and mediated model demonstrated good fit for the data at *χ*^2^ = 376.923, *p* < 0.01; CFI = 0.914; RMSEA = 0.073, 90% CI [0.066, 0.081]. Standardized coefficients for the structural paths are shown in Figure 5. PsyCap (*β* = 0.66, *p* < 0.001) and SWB (*β* = 0.26, *p* < 0.001) were positively associated with CC. Consistent with our prediction, PsyCap was positively associated with SWB (*β* = 0.42, *p* < 0.001); thus, H3 was verified. The indirect effect of PsyCap on CC *via* SWB was also significant (*β* = 0.11, 95% CI [0.09, 0.14]). Further, the total effect of PsyCap was significant (*β* = 0.77, 95% CI [0.70, 0.80]; Table 3). These findings indicated that SWB partially mediated the relationship between PsyCap and CC. Therefore, H4 was supported.

**Table 3:** Analysis of mediating effect of SWB on PsyCap between CC.

	*β*	SE	95% CI
Total effect	0.77^**^	0.02	[0.70, 0.80]
Indirect effect	0.11^**^	0.01	[0.09, 0.14]
Direct effect	0.66^**^	0.03	[0.57, 0.69]

*β, standard regression coefficient; SE, standard error; CI, confidence interval*.

** *p* < 0.01.

## Discussion

This study was conducted to examine both the mediating and moderating effects of SWB on CC, using a sample of preschool teachers. Results showed that SWB played a dual role of mediation and moderation in the relationship between PsyCap and CC, although such a situation is rare. Consistent with our predictions, model fits were also at or near predetermined standards, thus indicating that our results supported a framework in which SWB mediated and moderated the relationship between PsyCap and CC. Further, a Pearson correlation coefficient revealed a score of 0.68. Overall, SWB among this study’s preschool teachers did not seem robust (an average of 3.63), but PsyCap was found to be relatively strong (an average of above 4). As little research has been conducted on the relationships between PsyCap, SWB, and CC among preschool teachers, these findings can help educators both improve SWB and enhance work enthusiasm by developing positive psychological strength. Results indicated a close positive correlation between PsyCap and SWB in this context, thus supporting our hypotheses.

### Relationship Between Psycap and CC

Based on our full structural modeling analyses, we determined that there was a significant positive correlation between PsyCap and CC among preschool teachers. This finding is consistent with previous studies demonstrating that PsyCap serves as a psychological resource with positive impacts on CC, work performance, and attitude. Given that PsyCap influences attitudes in general, it is perhaps unsurprising that it also influences CC (Mortazavi et al., 2012; Jung and Yoon, 2015; Nielsen et al., 2016; Sinan, 2016; Lee et al., 2017; Singhal and Rastogi, 2018). This finding is important, however, because it demonstrates that preschool teachers with higher CC can help improve the overall standards of both early childhood education and care. A higher level of PsyCap increases an individual’s confidence and their efforts to succeed and achieve goals, and improves their ability to deal with difficult problems. PsyCap can be regarded as a positive factor in a person’s overall well-being. Individuals with a high level of PsyCap can better adjust to work pressure, maintain an optimistic attitude at work, and have hope for how their career will develop in the future, thus showing a high CC (Singhal and Rastogi, 2018). This further suggests that teachers with enhanced PsyCap will exhibit increased CC. As such, PsyCap interventions, such as those proposed by Lee et al. (2017), should be implemented to improve hope, resilience, optimism, and self-efficacy among preschool teachers.

### Psychological Capital Interacts With SWB to Predict CC

Our H4 was that SWB would moderate the relationship between PsyCap and CC; results supported this assumption. Specifically, this study introduced SWB as a positive psychological variable and further explored the mechanism underlying the relationship between job engagement and burnout. Here, SWB was positively correlated with all dimensions of job burnout. Indeed, higher SWB was directly associated with lower overall job burnout, which is consistent with previous findings (Goelman and Guo, 1998; Cenkseven-Onder and Sari, 2009; Wells, 2014). This study’s results also support the moderating model involving the effects of SWB on the relationship between PsyCap and CC. That is, SWB played a direct moderating role in this relationship.

The above findings suggest that PsyCap can effectively increase CC, but that SWB moderates this relationship, thus influencing how PsyCap shapes CC among preschool teachers. For instance, teachers with higher SWB exhibited higher levels of CC when compared to those with lower SWB. Further, the degree to which PsyCap increased CC among teachers with higher SWB was greater than it was among those with lower SWB. This finding also supports related research demonstrating that SWB plays a moderating role in this relationship. Our results suggest that, in the Chinese preschool setting, teachers with higher SWB possess both superior psychological resources and CC. In turn, this indicates that those with higher SWB may have greater amounts of working PsyCap, thereby allowing them to experience better long-term CC as a result of their inherent advantage in the preschool educational field. From a different perspective, teachers with higher levels of SWB may have deeper overall understandings of their schools, local cultures, and issues related to student parents, thus enabling them to deal with workplace challenges and solve associated problems more effectively than teachers with lower levels of SWB. This suggests that preschool teachers with higher overall SWB should be encouraged to share advice with their peers through empowerment exercises in order to improve general PsyCap and CC levels. This will be of value when teachers are developing personal tacit knowledge or practical intelligence involving elements of hope, resilience, optimism, and self-efficacy.

High SWB is also effective for helping individuals realize the related endogenous mechanisms of adversity and/or stress by triggering positive emotions, thus enriching their perspectives on problem-solving and generating a diversified range of possible solutions (Brickman et al., 1978; Lee et al., 2017). Higher SWB is also associated with increased income levels, better health, higher job satisfaction, positive general attitudes toward life, more vitality, and higher self-efficacy (Graham et al., 2004; De Neve and Oswald, 2012). As such, PsyCap has a range of different impacts on preschool teachers. Further, these may manifest differently according to individual SWB levels. Different from the findings of a study by Panaccio and Vandenberghe (2009) indicating that both NC and perceived sacrifice as associated with job turnover were unrelated to well-being, our findings directly indicated that self-efficacy and optimism interacted with SWB in predicting individual levels of NC and AC. Meanwhile, optimism was shown to interact with SWB in predicting CC. These results indicate that greater importance should be attached to both AC and NC when attempting to improve CC among preschool teachers. General society should truly respect teachers by attaching greater importance to their profession, in such a way that it improves overall social status for these individuals. For instance, government education departments and preschool administrations should give teachers more autonomy when conducting their work, allowing them appropriate decision-making autonomy and promote feelings of value through work-related encouragement. In this way, teachers can achieve greater motivation, more enriching experiences, and increase their levels of self-efficacy. Preschools should also establish effective teacher evaluation mechanisms, create fair competition environments among its employees, pay increased attention to expressions of both high and low self-efficacy, and facilitate high workplace optimism. On the other hand, preschool teachers should optimize their teaching staff by matching them to their respective positions in a way that appropriately reflects their abilities, certifications, and training. These teachers should also make practical career development plans that promote both professional growth and self-development. Their work needs should thus be met so that they can not only understand their own levels of performance and experience the pleasures of career success, but also develop positive career expectations, enhance their teaching effectiveness, and maintain the necessary motivation for sustainable growth. These proposals should ultimately be effective in enhancing overall levels of CC.

### Subjective Well-Being Mediates the PsyCap-CC Relationship

H3 posited that SWB would be positively associated with CC. SWB is a positive emotion which relates to life satisfaction and mental health. These factors have a positive effect on work attitude and performance (Jothimani, 2016; Satoh et al., 2016). In addition, people’s intrinsic values that give meaning to life govern their motivation to work (Siwek et al., 2017). Preschool teachers with high SWB have strong work enthusiasm and often feel happy and satisfied with the teaching work they are engaged in. Therefore, they can work with a positive and optimistic attitude which improves the quality of teaching services and increases children’s positive responses to the teachers, thus heightening their CC.

According to H5, SWB would mediate the relationship between PsyCap and CC in such a way that it would significantly diminish if SWB were controlled. As such, this study introduced SWB as a positive psychological variable to further explore the mechanisms underlying the relationship between PsyCap and CC. Further, SWB was positively correlated with all CC dimensions. Consistent with previous research, this study found that preschool teachers with higher SWB had higher CC (Siu, 2013; Li et al., 2014; Saraf, 2017). This also supports the intermediary model of SWB regarding the relationship between PsyCap and CC, indicating that PsyCap may directly affect both increases and decreases in CC. This can also be evident through improved SWB, which indicates that SWB directly affects the relationship between PsyCap and SWB as well as the related interaction mechanism.

Preschool teachers with higher levels of PsyCap also experience higher levels of CC. This is partly because these individuals also tend to experience higher SWB when compared to those with lower levels of PsyCap. Further, SWB partially mediated the relationship between PsyCap and CC, thus supporting H5. This relationship between CC and SWB has led researchers to establish a measurement tool for measuring career exploration (Peterson, 2012). Some studies have thus found that employees with better quality of working life (QWL) more effectively managed customer complaints, thereby suggesting that QWL may also partially mediate the relationships between PsyCap and work outcomes (Kim et al., 2017; Kafashpoor et al., 2018). In addition, Negar et al. (2015) found that QWL mediated the relationship between PsyCap dimensions and life satisfaction. Research has also shown that CC is positively associated with work outcomes as well as both job and career satisfaction (Avey et al., 2010; Shah, 2011; Khan, 2013; Battu and Chakravarthy, 2014; Bianchi, 2015). Many studies on QWL have also found that it is positively associated with career satisfaction (Nabati et al., 2014; Agarwal et al., 2019).

The results are not only consistent with the broaden and build theory and SDT proving that the PsyCap will successfully predict SWB and CC of the employees because of the positive emotions of hope, efficacy, resilience, and optimism, but also empirically assert that the SWB will act as a partial mediator between PsyCap and CC. As this study’s findings provide support for the partial mediating effects of SWB on the relationship between PsyCap and CC, they are valuable additions to the current literature on positive psychology, which continues to interpret positive organizational behavior from the viewpoint of Chinese culture.

### Limitations

Although it produced valuable findings, this study had some limitations. First, surveys were distributed *via* e-mail and there was a relatively low response rate (80%). As already mentioned, there was also a lack of previous research concerning the study topic. Further, all survey data were self-reported, potentially introducing subjective predictive validity. Additional research is thus needed to verify this study’s findings. Future related studies should combine an assortment of other assessment mechanisms in approaching the issue. This includes interviews, observations, and teacher ratings in order to overcome the limitations associated with self-reported data. The study sample was also derived from a total of 41 urban preschools in Wuhan, which, as a new first-tier city, has less universality. Although Wuhan residents enjoy relatively convenient, comfortable, and enriching lives, they have also faced the problems of high housing prices and costs of living for a long time. As such, the general Wuhan lifestyle may not be reflective of that in other regions. Because of this, regional differences and limitations resulting from the small sample size may limit the generalizability of this study’s results. Larger national studies are thus needed to provide a basis for comparison.

### Conclusion

The analysis results produced by this study directly showed that PsyCap and CC were two independent, but interrelated, psychological states. Findings also indicated a significant positive correlation between PsyCap and CC, and that when PsyCap increased, CC tended to increase. Further, SWB played a partial regulatory role in the relationship between PsyCap and CC. Correlation analyses also revealed that PsyCap and CC were positively related and that SWB mediated and moderated this relationship. As the literature concerning the interactions between PsyCap, SWB, and CC among urban preschools teachers is relatively new and developing, these issues are still poorly understood in the Chinese cultural context. However, this study’s findings provide a valuable evidence-based foundation for designing prevention and intervention efforts aimed at increasing the positive impacts of PsyCap on CC. Results further suggest that it is essential to focus on both PsyCap and SWB, when attempting to improve CC in the educational instruction context.

## Data Availability Statement

The datasets generated for this study are available on request to the corresponding author.

## Ethics Statement

The studies involving human participants were reviewed and approved by the Research Ethics Committee of the Hubei University of Education. The patients/participants provided their written informed consent to participate in this study.

## Author Contributions

YG has made substantial contributions to the design of the work and the analysis of data for the work. LC has made substantial contributions to the introduction and literature review. All authors contributed to the article and approved the submitted version.

### Conflict of Interest

The authors declare that the research was conducted in the absence of any commercial or financial relationships that could be construed as a potential conflict of interest.
